# Performance Evaluation of Multi-UAV Network Applied to Scanning Rocket Impact Area †

**DOI:** 10.3390/s19224895

**Published:** 2019-11-09

**Authors:** Maurício R. Silva, Elitelma S. Souza, Pablo J. Alsina, Deyvid L. Leite, Mateus R. Morais, Diego S. Pereira, Luís B. P. Nascimento, Adelardo A. D. Medeiros, Francisco H. Cunha Junior, Marcelo B. Nogueira, Glauberto L. A. Albuquerque, João B. D. Dantas

**Affiliations:** 1 Department of Computer Engineering and Automation, Federal University of Rio Grande do Norte, Natal 59078-970, Brazil; deyvidlucas@ufrn.edu.br (D.L.L.); mateusrodrigues@ufrn.edu.br (M.R.M.); diego.pereira@ifrn.edu.br (D.S.P.); lbruno@ufrn.edu.br (L.B.P.N.); adelardo@dca.ufrn.br (A.A.D.M.); 2 Federal Institute of Paraíba, Sousa 58805-345, Brazil; 3 Sidia Institute of Science and Technology, Manaus 69050-020, Brazil; 4 Federal Institute of Rio Grande do Norte, Parnamirim 59143-455, Brazil; 5 School of Science and Technology, Federal University of Rio Grande do Norte, Natal 59078-970, Brazil; heliojunior@bct.ect.ufrn.br (F.H.C.J.); marcelonogueira@ect.ufrn.br (M.B.N.); 6 Barreira do Inferno Launch Center, Parnamirim 59140-970, Brazil; leilsonglaa@fab.mil.br (G.L.A.A.); dolvimjbdd@fab.mil.br (J.B.D.D.)

**Keywords:** ad hoc network, wireless sensor networks, FANET, multi-UAV system monitoring, communication architecture, network performance

## Abstract

This paper presents a communication network for a squadron of unmanned aerial vehicles (UAVs) to be used in the scanning rocket impact area for Barreira do Inferno Launch Center—CLBI (Rio Grande do Norte, Brazil), aiming at detecting intruder boats. The main features of communication networks associated with multi-UAV systems are presented. This system sends information through Wireless Sensor Networks (WSN). After comparing and analyzing area scanning strategies, it presents the specification of a data communication network architecture for a squadron of UAVs within a sensor network using XBee Pro 900HP S3B modules. A brief description is made about the initial information from the construction of the system. The embedded hardware and the design procedure of a dedicated communication antenna to the XBee modules are presented. In order to evaluate the performance of the proposed architecture in terms of robustness and reliability, a set of experimental tests in different communication scenarios is carried out. Network management software is employed to measure the throughput, packet loss and other performance indicators in the communication links between the different network nodes. Experimental results allow verifying the quality and performance of the network nodes, as well as the reliability of the communication links, assessing signal received quality, range and latency.

## 1. Introduction

Unmanned aerial vehicles (UAVs) have been widely used in a large number of applications over the last years in many different fields, for example, environmental monitoring [1,2,3], visual coverage [4,5,6], natural disaster management [7,8,9,10], among others.

When scanning large areas, a multi-UAV system can execute a monitoring task more efficiently and faster than a single aircraft, with the possibility to acquire images simultaneously and from different points of view. A team of UAVs working according to an adequate collaboration strategy can overcome typical limitations in terms of autonomy and short-range associated with a single UAV, therefore covering larger areas and ensuring the fast execution of the mission, while also aggregating cost reduction, fault tolerance and scalability to the task [11].

Establishing a stable and reliable UAV network is a challenging task. Many issues should be addressed before its effective use in order to provide efficient communication among the UAVs. Differently from common ad hoc networks, flying ad hoc networks (FANETs) must deal with several hard constraints depending on the application, including high mobility and varying speeds of the UAVs, intermittency of communication links and dynamic changes in topology [12]. In order to preserve the quality of services and ensure the reliable transmission of packets between end nodes, routing protocols must address specific issues other than those found in conventional proactive or reactive schemes. The network must reorganize itself often and, sometimes, it may even get partitioned. Since autonomy is an important issue in UAVs, FANETs must deal with energy saving in order to increase the network lifespan [13]. Therefore, all components of the FANETs face distinct challenges to be solved. Some recent works have addressed improvements in the communication system through routing protocols [14,15], Quality of Service (QoS) [16], distinct architectures [17,18], resilience [19], as well as other characteristics that may bring additional reliability and safety to the system.

Barreira do Inferno Launch Center (CLBI, *Centro de Lançamento da Barreira do Inferno*) is one of the Brazilian suborbital rocket launch centers, which is located in the city of Parnamirim, state of Rio Grande do Norte at the northeastern shore. During the launching chronology, before a rocket launching is performed, it is necessary to scan the predicted impact area in order to detect possible intruder boats and escort any ship found out of the restricted area. Nowadays, the impact area scanning procedure is executed by the CLBI using a manned aircraft (model EMB-110 Bandeirante), which involves a time consuming and expensive procedure. This task can be more efficient and cost-effective if performed by UAVs [20].

**Figure 1:**
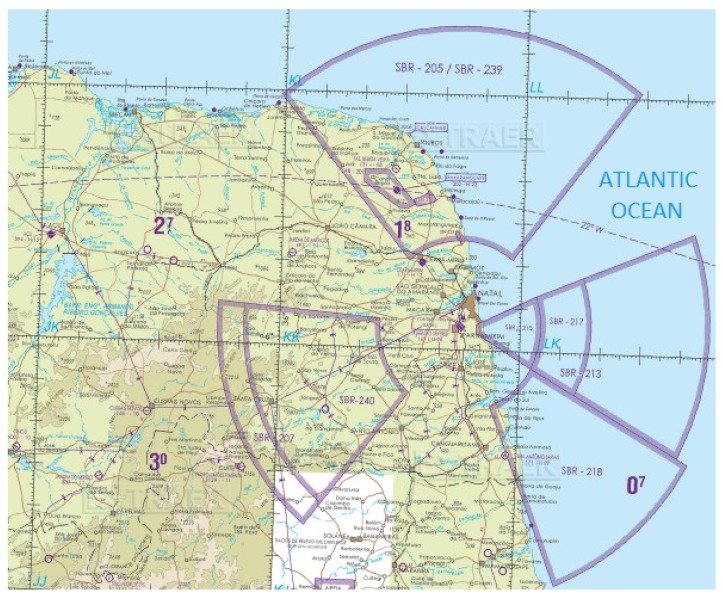
Impact area of a rocket launched from CLBI (Barreira do Inferno Launch Center).

For basic training rocket and intermediate training rocket launchings, the rocket reach and radius of the impact area are within the range of a few kilometers, as it can be seen in Figure 1, where SBR-215 and SBR-217 correspond to restricted airspaces. The scanning procedure can be performed by a single UAV in this case [20,21]. For larger rocket launching procedures, the reach and radius of the area to be scanned is also larger according to Figure 1, where SBR-213 is a restricted airspace. A single UAV will not be capable of performing the scanning procedure accurately, mainly due to time constraints. In such cases, a multi-UAV system composed of autonomous aerial vehicles is preferred instead due to their inherent ability to perform the scanning procedure in a fast and economically efficient way.

When scanning the rocket impact area with a multi-UAV system, some aircraft may fly beyond the signal range of the base station (BS) antenna. In this case, some kind of communication infrastructure is required to transmit information between the distant aircraft and the BS. The data transmission system must be capable of sending telemetry messages associated with the swept area, while also informing if any ship is found and, if so, sending an image of the target to the BS. Since the rocket impact area is offshore, that is, in the Atlantic Ocean, the communication infrastructure can be provided by the aircraft themselves by establishing a FANET among the UAVs. This work aims to develop and validate the performance of a FANET architecture. It intends to ensure the transmission of control data and images from ships located in a restricted offshore area using a network composed of ZigBee sensors embedded in a UAV squadron. The aircraft are responsible for scanning the impact area of rockets launched offshore by the CLBI so that the eventual presence of intruders is detected efficiently and reliably in a less time-consuming and low-cost approach.

Some works, for example, Nasution et al. [22] present the design, communication protocol and testing procedure of a system for sending UAV telemetry messages to a ground control station (GCS) using ZigBee protocol. Zhou et al. [23], Vale [24] proposes a cooperative aerial-ground vehicular network architecture, which employs ZigBee to send telemetry messages and a Wi-Fi network to send images aiming at disaster rescue missions or area investigation. Ahn et al. [7] designed a UAV communication protocol for Wi-Fi networks based on time division multiple access (TDMA) transmission at the network layer. Besides, due to delayed packet delivery issues, the use of a model that supports a guaranteed time slot (GTS) is suggested based on the ZigBee protocol associated with the network link layer. Bacco et al. [25] proposed a testing platform relying on IEEE 802.15.4-based communication between UAVs for exchanging data with ground sensors to monitor and control automated crops. Braga et al. [26] considered the use of unmanned robotic vehicles in maritime operations, where the main communication restrictions between the vehicles and mission controller are associated with long distances and/or low-power transmissions. In this context, it is reasonable to state that the aforementioned works have used Wi-Fi to transmit images, as well as ZigBee or Wi-Fi protocol to send telemetry data between the UAVs and the base station. The particular difference of the proposed study lies in the development and test of a communication protocol to be embedded in a Multi-UAV system to transmit images and information in a real-time approach from UAV to UAV (U2U) based on XBee sensors that employ ZigBee protocol. The tests are performed on quadrotors model DJI Phantom 3. The multi-UAV communication system is embedded in the aircraft by their respective manufacturer [27], considering that the UAVs are responsible for sweeping rocket impact areas on launch missions aiming at the automatic detection of ships by aerial image processing. In offshore areas, it is not possible to use the Wi-Fi protocol due to limited area range and mobility between nodes of the aerial network.

The ZigBee protocol is used by Mushtaq et al. [28], Min and Nam [29] to perform fly-by-sensors (FBS) flight control with stable flight formation, while Sineglazov and Daskal [30] propose a UAV navigation system for dense urban areas in tunnels or indoor environments. The flight planning and formation model is discussed in Lee et al. [31], Park et al. [32] where an effective routing protocol for navigation control and an information sharing system with onboard sensors and flight path formation algorithms are proposed for various UAVs. Popescu et al. [18] propose an optimized trajectory design to avoid restricted regions aiming at bypassing them through close preset landmarks. This approach ensures a proper communication time to minimize the path length and discuss a method for locating and grouping sensors in an optimal coverage, also taking into account communication information between UAVs. Guillen-Perez and Cano [33] freview proposed mobility, positioning and propagation models for FANETs. This work highlights that the common limitation that affects such aspects is the lack of studies that assess the influence of UAVs on embedded communication devices. For this reason, it measures the impact of a UAV on the onboard Wi-Fi radiation pattern.

In this work, the aircraft used to scan the area presents a flight range of up to 20 h. Two distinct man-machine-Interface (MMI) approaches are used: remote pilot interface and operator interface (just in case of emergency). As for the communication link for flight control and navigation messages, two different link radios are used: a 100-km range very-high frequency (VHF) uplink and S-band downlink to receive telemetry data and images from a single UAV as recommended in [20]. In order to perform U2U communication, ZigBee devices that work with an operating frequency of less than S-Band were chosen so that the signal suffers fewer attenuation effects and low manufacturing cost is obtained.

The main contribution of this paper can be summarized as follows:

- Based on the analysis of the various possible configurations of the communication network and the specific features to perform the area scan over the sea, an appropriate architecture is proposed for this particular application based on devices that support IEEE 802.15.4 standard and ZigBee communication protocol. This solution is capable of sending telemetry data and images between the UAVs using a FANET;
- A communication protocol is proposed for a FANET operating in the maritime area, being capable of transmitting telemetry images and data. Due to the particularities of the scenario and involved application, XBee Pro 900HP S3B sensors were adopted. Besides, the system meets the following requirements:

The UAVs send telemetry data on the scanned area and target locations periodically using control messages.

If the image system embedded in a UAV locates a target, the communication system must send the image to the HUB node through the FANET, which sends it to the GCS. Due to the restrictions of the offshore area and the payload of XBee sensors, it is necessary to fragment the image, send the packets in fragments, perform the control of the image reception and, if necessary, request the retransmission of some lost packet. For this purpose, data and confirmation messages are employed. Thus, the GCS confirms to the UAV through a message that the image was received.

- The communication protocol was validated by assessing different scenarios and controlled environments. The first scenario validates the wireless sensor network (WSN) throughput on a mesh network. The purpose is to check whether ZigBee sensors meet image system requirements to send messages according to the time required to sweep the area. The second scenario assesses the fault tolerance capacity of the ad hoc network to reorganize itself into a multi-UAV system. If an intermediate node fails, the network reconfigures itself by finding new routes in the FANET in all test conditions;
- Simulation tests were performed to define a position for which the aircraft structure influences as little as possible the electromagnetic propagation of the antenna used by XBee sensors. The omnidirectional aspect of the antenna is then maintained and the surrounding UAVs will have nearly the same signal strength if the distances are equal;
- A channel based on Rice propagation model using line-of-sight (LOS) was analyzed by simulation employing a synthetic signal associated with XBee devices to define how farther one aircraft can stay away from another while maintaining minimum QoS, that is, to ensure that the signal strength at reception has a power exceeding −100 dBm;
- Tests were performed in a controlled environment to transmit images between the multi-UAV network and the base station, where the image is split into multiple packets. To simulate the tests, XBee sensors embedded in the UAV model DJI Phantom 3 are employed.

A preliminary version of this work was published as a conference paper in [34]. The present extended version of this study includes the area scan strategies detailed as follows. In order to compare them properly and define an appropriate architecture for the application, an analysis of possible network characteristics for multi-UAV systems is carried out. A detailed description of a FANET developed for a multi-UAV system can then be applied to the detection of intruder boats in the predicted impact area of suborbital rockets launched from the CLBI. In this context, the scanning procedure can be performed in an efficient and reliable manner, thus reducing cost and time.

The remainder of this paper is organized as follows. Section 2 presents the main characteristics of multi-UAV communication systems. The scanning strategies are presented and discussed in Section 3. Section 4 describes the multi-UAV system, the image processing system, as well as the proposed network architecture for the addressed application in terms of the software and hardware required to support the network. The scenarios for which the tests are carried out are described in Section 5. Section 6 presents the results of tests performed on a data transmission network with ZigBee devices. The main conclusions are given in Section 7.

## 2. Communication Networks for UAVs

Ad hoc network does not rely on a pre-existing infrastructure, while communication among the nodes occurs without any centralized administration. Common instances of ad hoc networks are mobile ad hoc networks (MANETs), vehicular ad hoc networks (VANETs) and FANETs [13].

However, unlike MANETs and VANETs, FANETs have specific features and requirements associated with their main applications, which may involve a dynamic behavior of the network nodes. Some details on FANETs are presented in the next subsection.

### 2.1. FANET

A FANET consists of a group of homogeneous or heterogeneous flying agents that are capable of communicating with each other as a team, interacting with their neighboring to acquire valuable information [35]. The flight speed can be in the range from 0 to more than 100 m/s with two-dimensional (2D) or three-dimensional (3D) movements, which are commonly controlled according to the mission. The topology changes can be stationary, slow or very fast, with disorderly or synchronized movements depending on the objective [13].

In a FANET, the network architecture design takes into account a routing protocol with the capacity to reorganize itself in the case where one or more UAVs fail. Protocols with proactive or reactive routing algorithms may not work as a consequence of low processing capacity and low memory of the nodes. When such factors exist, the FANET must be capable of forwarding packets between the source and destination of the nodes, thus optimizing the chosen metric and incorporating mechanisms to save energy and maximize the network lifespan.

#### 2.1.1. Characteristics of FANET

An essential characteristic of a FANET is that the communication among UAVs must be implemented through an ad hoc network which does not fully depend on infrastructure connections. This network works through the collaboration and coordination among the nodes. Otherwise, it cannot be categorized as a FANET [11].

**Figure 2:**
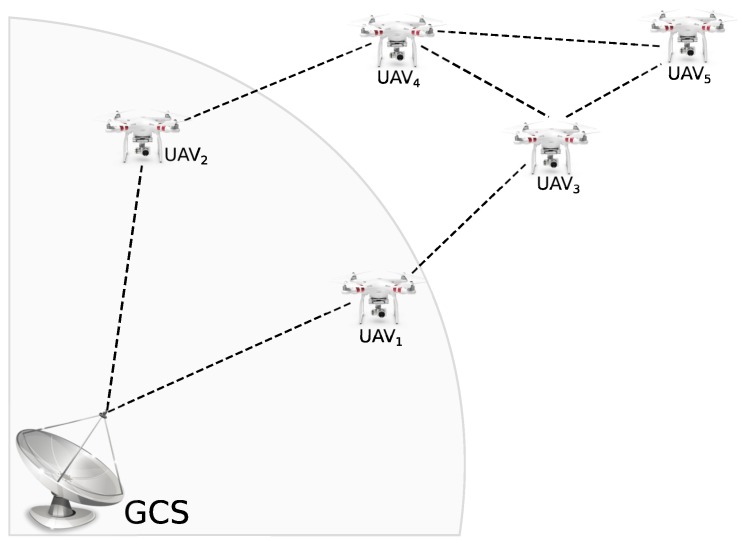
Simple example of a FANET (Flying ad hoc network).

Figure 2 represents a simple composition of a FANET. The ad hoc network is composed of five UAVs and one ground control station (GCS). In this case, UAV1 and UAV2 have one direct link with GCS. The remaining ones need to use them as routers to send or receive information from the GCS. The network topology is dynamic and can be changed at any moment but it is absolutely necessary that at least one UAV provides connectivity with the GCS.

#### 2.1.2. Main Applications

FANETs can be applied to some specific situations, for example, UAV network to provide an internet access infrastructure, UAV network for localization and attack for military purposes and UAV network for remote sensing or scanning. In the first case, the UAV network is supposed to be used as an internet access point. This type of network requires slow movement of the UAV. The communication infrastructure must be stable and capable of supporting a high throughput and access [36]. When used for localization and attack for military purposes, UAV networks must be designed so that the node is capable of recognizing a target and executing the attack, with the possibility of leaving the area immediately after the task execution; or performing self-detonation to eliminate the enemy. The goal is to search for a target in a way that is not necessary to complete scan the area as a whole [37]. When UAV networks are designed for remote sensing and scanning specific areas, using computer vision techniques makes it possible to scan the complete area under monitoring [38].

#### 2.1.3. Routing Strategy

According to Gupta et al. [13], a FANET architecture is directly related to the objective of the application, the flight plan and the path associated with the aircraft. After such priorities are defined, a routing strategy can be chosen. A network where the routing is performed by aircraft must take into account the following aspects in order to offer high quality services: location of nodes, energy saving, good robustness to intermittent links and topology changes.

For a FANET which aims at scanning a specific area, it is possible to define an appropriate routing protocol because the main characteristics are known, such as the synchronized movements and constant velocities of the nodes. Moreover, resources such as storage, signal strength and throughput are limited. The number of hops, interference and network conditions depend on the distance and movements of the nodes, which also affect the bandwidth limit. Zafar and Khan [39] present a study about routing protocols applied to FANETs, where the following protocols can be considered more important: optimized link state routing protocol (OLSR), destination-sequenced distance-vector routing (DSDV), ad hoc on demand distance vector (AODV) and better approach to mobile ad hoc networking (BATMAN).

Ruan et al. [40] discuss how information interaction among UAVs plays an essential role, while the communication mission involving data sharing, transmission and retransmission can be carried out cooperatively. In this sense, this work focuses on performing simulations on the efficient transmission of information and ensure the quality of UAV communication. Hong and Zhang [15] is based on the rapid change of topology that affects the performance of routing protocols in FANETs to propose an appropriate routing scheme in complex scenarios. Furthermore, a suitable routing protocol is selected for maintaining network performance at a high level. The concerned performance metrics are packet delivery ratio, network throughput, average end-to-end delay and average jitter.

### 2.2. Parameters for Measuring the Performance and Quality of the Data Transmission Network

Perdana and Wibowo [41] define QoS as the speed and reliability level of delivery from various types of data loads in a communication network. This parameter indicates the ability of the network to provide better conditions for the traffic service. Several metrics are necessary to obtain QoS from networks, for example, delay, throughput and the number of lost packets.

#### 2.2.1. Delay

Delay or latency through wireless networks is the time interval required by the packets to propagate from the source to the destination. The end-to-end packet delay is made up of the summation of route discovery (source-processing delay), queuing (network delay) and propagation and transfer time (destination delay). Many applications require an end-to-end delay guarantee for time sensitive data [42]. Equation (1) expresses the delay (σ) as the average difference between a number of delivered data (η) and the throughput (τ) [41].

(1) $$\sigma =\frac{\eta }{\tau }$$

#### 2.2.2. Throughput

Moridi et al. [42] define the throughput as the ability of data packets to be successfully sent from the source node to the destination one in the time unit. In this study, this measure is given in bits per second and calculated as Equation (2):

(2) $$\tau =\frac{8\;Tps}{Tlps-Tfps}$$

where Tps is the total number of sent bytes and Tlps, Tfps are the time instants at which the last and the first packets were sent, respectively.

#### 2.2.3. Packet Loss

Packet loss is the number of failed packets in the delivery process through the transmission media. Different factors can contribute to this issue, such as signal degradation in network channels, corrupted packets and failures in the network device or in the network routing process. The packet loss is calculated by Equation (3),

(3) $$\zeta =\frac{\alpha -\beta }{\alpha }100\%$$

where ζ is the packet loss percentage, α is the number of sent packets and β is the number of delivered packets [41].

#### 2.2.4. Received Signal Strength Indicator (RSSI)

The RSSI is a radio frequency power indicator that reaches the input of the receiving antenna through a channel. It is considered an important measurement for maintaining a good quality of the data sent from the UAVs to the ground station. In this situation, the aircraft is exposed to some physical effects, such as large-scale and small-scale fading that can decrease the signal quality when reaching the receivers on the ground. Therefore, if the power is less than the receiver threshold, the data received will be more difficult.

To ensure that the power at the receiver is higher than the minimum allowable value and the throughput rate as defined by the device datasheet, it is essential to calculate the maximum distance that the UAVs must maintain from each other, also considering the received power of the radiofrequency signal. Based on Equation (4), it is possible to determine the signal strength as a function of the distance between the UAV and/or ground base station. This equation can be properly modified by adding a random factor $X_{\sigma }$ associated with the environmental obstacles to model the normal log shading since such effect affects the average signal strength on a large scale [43].

(4) $$P_{Rx}=P_{Tx}+G_{Tx}+G_{Rx}+20log_{10}\left(\frac{\lambda }{2\pi d}\right)+X_{\sigma }$$

where $P_{Tx}$ is the device transmission power; $G_{Tx}$ and $G_{Rx}$ are the gains of the transmitter and receiver antennas, respectively; *d* is the distance between transmitter and receiver; and λ is the wavelength [26].

## 3. Strategies for Scanning the Impact Area

Due to security issues, for every ballistic rocket launched towards the sea, a foreseen impact point and trajectory are determined in order to establish an exclusion area both in the air and sea where there is a significant probability of impact. In this section, two scanning strategies are proposed for a multi-UAV system aiming at the exclusion area surveillance in such a way that the area can be checked while ensuring both minimal energy consumption of the UAVs, as well as stability and reliability of the network connecting the aircrafts.

When establishing the appropriate scanning strategy, there are some aspects regarding the scanning purpose that must be taken into account. For instance, one of such requirements may include providing an aerial image of the entire scanning area. Therefore camera aperture, altitude and flight speed are key examples of relevant parameters for planning the mission. Besides the aspects that are inherent to the task to be executed, there are also other important considerations related to the resources required for executing the mission. Thus, a good scanning strategy should define routes that ensure minimal energy consumption in order to provide maximum flight autonomy, as well as keep the aircrafts in close proximity to ensure a stable communication link between them, which further minimizes the probability of failure.

For the specific application analyzed in this paper, the impact area may vary from a few to hundreds of square kilometers and, depending on the rocket itself, the area of impact can be located close to the coast or a few hundred kilometers offshore. In this scenario, the group of UAVs to be assigned to the mission should be as small as possible to ensure at least four hours of flight autonomy. At least one of the aircrafts must be capable of keeping a reliable communication link with the required bandwidth and also the ability to communicate with the BS from any point in the impact area.

When the area to be covered is small, a single UAV can be assigned to the mission [21]. In this case, two simple strategies are found in the reference literature: spiral and back-and-forth methods [44].

### 3.1. Scanning Strategy Using Multi-UAV System without Area Decomposition

**Figure 3:**
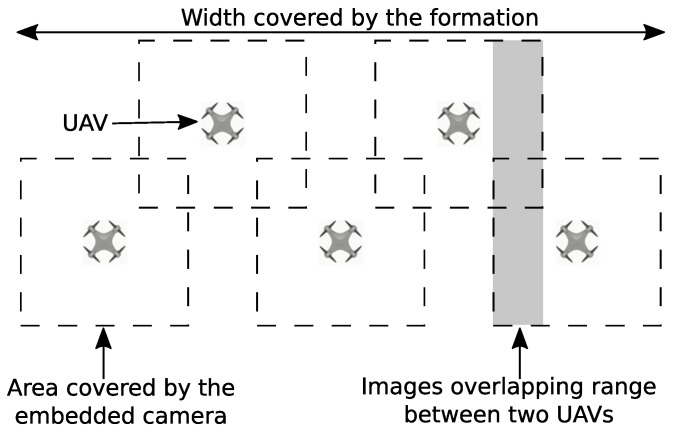
Flight formation for spiral scanning.

In the strategy proposed in this work, the UAVs should fly information in a side-by-side approach, while scanning the area as shown in Figure 3. In this scenario, the UAVs maintain the same distance between each other. The images are acquired periodically as a mosaic, which has the size of the coverage area of the camera embedded on the aircrafts times the number of UAVs in the network. Thus, it is possible to estimate the area shared between neighbors due to the overlapping, which is necessary to ensure that the impact area is properly scanned. In this scanning strategy, the distance between the two UAVs is determined to ensure the correct image superposition, which depends on the camera aperture and flight altitude. This kind of formation eliminated redundant links and, typically, the distance between two neighboring UAVs is shorter than the maximum range for the communication system embedded on the aircrafts. Therefore, this method will tend to provide more reliable communication.

**Figure 4:**
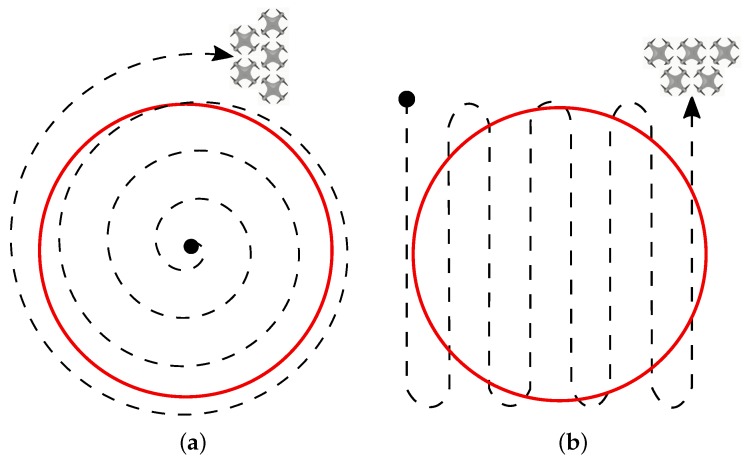
Area scanning strategies for the formation of unmanned aerial vehicles (UAVs). (**a**) Spiral pattern; (**b**) Back and forth pattern.

Applying the algorithm for spiral scanning as described by Öst [44], it is possible to keep the UAVs in the formation and as close to each other as possible. An alternative to the strategy lies in the back-and-forth methodology, where the inversion occurs in both directions, resulting in almost the same trajectories for all nodes. The spiral and back-and-forth methods are shown in Figure 4a,b respectively.

Fully scanning an area while maintaining all UAVs in a fixed formation is useful for situations where the processing speed and time for mission completion are the main factors to be considered. In this case, at least one UAV capable of maintaining a reliable communication link with the base station throughout the whole scanning area is a mandatory requirement. Moreover, the communication link must ensure that there is enough bandwidth to intermediate the communication between other nodes with the base station. In this manner, the network can be established using the node with the widest range as a hub for the neighbors that are unable to communicate directly with the base station. In the case of a node failure, the neighboring nodes must reorganize themselves to maintain the formation. If a single node has the requirement to work as a hub, its failure will compromise the whole system as a consequence. Thus, it is recommended to use more than one hub in the network to provide redundancy and mitigate failure.

### 3.2. Scanning Strategy Using Multi-UAV System with Area Decomposition

**Figure 5:**
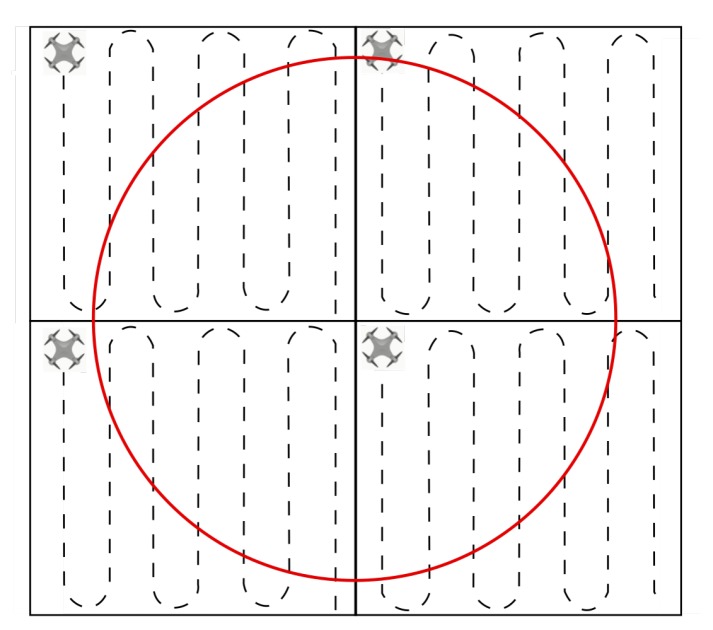
Area decomposed into four subareas by using the back-and-forth pattern.

When the communication between the base station and the farthest node becomes unreliable due to distance, the solution is to subdivide the scanning area into subareas and assign each one of them a UAV, thus establishing a mesh network among the aircrafts. This allows the information to jump from one node to another until it reaches the destination, for example, the base station. Figure 5 represents the aforementioned method, which leads the system range to increase. Although the aircraft is distant, it can be coordinated in a synchronous flight formation when using a strategy that scans subareas with the same size. The size of each subarea must be defined so that each UAV has a reliable communication link with its neighboring units.

A relevant aspect of this application is that obstacles do not exist within the scanning area since the aircrafts travel over the sea, thus simplifying the trajectory planning. When one of the nodes fails, areas that have already been scanned should not be rescanned. In the case of a node failure, the method proposed by Marro and Goncalves [45] could be adopted to subdivide the unscanned area into new subareas, also taking into account the spaces that have been scanned so far. Yet there is the need for an algorithm capable of forwarding data packets among all UAVs in the system to the base station in a reliable manner, thus ensuring that the communication remains established.

### 3.3. Comparative Analyses among Scanning Strategies Using Multi-UAV Systems

In order to determine which strategy provides the shortest scanning time for the area of impact, while simultaneously ensuring a reliable network for exchanging data among UAVs and base station, both proposed strategies will be compared as detailed in Table 1.

For both of them, the scanning time is proportional to the number of UAVs assigned to the mission. The maximum size for the searched area when the subdivision is not applied is restricted by the range of the node that is leveraged as the hub. When subdivisions are applied, the size of the scanning area can be larger than that in the first scenario, since the information can be routed from a distant aircraft to the base station through a mesh network. Data is sent from one node to another until it reaches its final destination. When the area is not subdivided, the system relies on a dedicated and robust link that connects the hub to the base station. The remaining UAVs communicate with the hub node through a network configured in a star topology coordinated by the hub. Alternatively, in order to increase the reach, it is possible to establish a mesh network among some of the nodes if they are not able to communicate directly with the hub.

In the scanning method without subdivision, since the nodes are close to each other, the required time interval for reorganizing the network is shorter when one node fails, where the formation is simply reorganized. Besides, the energy consumption due to signal transmission is reduced in this case, implying the extension of the system’s lifespan. When a subdivision is used instead, as the nodes are farther from each other and, if failure happens to occur, the unscanned area must be divided again among the remaining nodes. Due to the greater distance between nodes, more power will be required by the communication system.

Considering the network behavior for each proposed strategy, as well as the specific characteristics of the addressed application where few nodes are available, a scanning strategy without subdivision was adopted at the first moment. This is due to the limited number of available UAVs embedded with a robust and long-range communication system. Based on the chosen strategy, it is possible to plan a set of experiments and establish metrics in order to measure network parameters, such as the received signal strength indicator (RSSI), latency, transmission rate, packet loss rate, jitter and network overhead, aiming at analyzing the network performance in terms of reliability and capacity of reorganization when adding or removing nodes.

## 4. Proposed System

**Figure 6:**
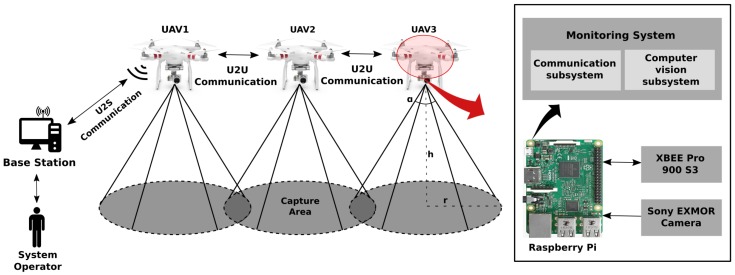
Architecture for the Multi-UAV system.

The proposed multi-UAV communication system is associated with a Penguin B aircraft fleet [20]. XBee sensors embedded in Raspberry Pi board perform U2U communication, up to the UAV hub. The FANET hub node performs UAV-to-BS (U2S) communication so that information is transmitted to the GCS. The network architecture, hardware, software and telemetry image and data transport protocol used are tested on small-scale four-wheel air vehicles. Figure 6 represents the testing architecture for the multi-UAV system. The next section is supposed to present a description of each subsystem.

### 4.1. Communication Subsystem

The communication network of the proposed system is composed of a small number of aircrafts. In order to simplify the network architecture, the scanning strategy adopted without area subdivision is relatively simple and somehow sacrifices the efficiency of the scanning process, which could result in longer scanning time for a fixed area due to the overlapping approach used in image capture. However, this practice leads to improved security while sweeping the area. The UAVs have a coordinated flight course, running similar routes at a constant speed, as it is possible to determine future points and predict the network course. Thus, the proposed scanning strategy allows setting a flight plan that minimizes the distance between network nodes to avoid signal loss problems and the use of optimized end-to-end delivery techniques with a unicast packet.

Bearing this in mind, two types of communication protocols are used, that is, U2U and U2S. The U2U protocol is capable of performing the communication between the UAVs that compose the aircraft squadron in flight, thus allowing for the collaborative work. The U2S protocol is responsible for ensuring data exchange between the ground BS and the UAVs network.

#### 4.1.1. Hardware Specification

For testing purposes, the multi-UAV system network is physically implemented in XBee Pro 900HP S3B modules connected to Raspberry Pi type computers with Linux operating system, embedded in a quadrotor UAV type Phantom 3 equipped with Global Positioning System (GPS), autopilot and maximum flight time of about 25 min. The UAV model characteristics are: maximum take-off weight (MTOW) of 21.5 kg; the payload of 10 kg; fight autonomy of over 20 h (with a full tank of 7500 cc; environment protection against rain and snow; embedded power generator rated at 80 W [27].

**Figure 7:**
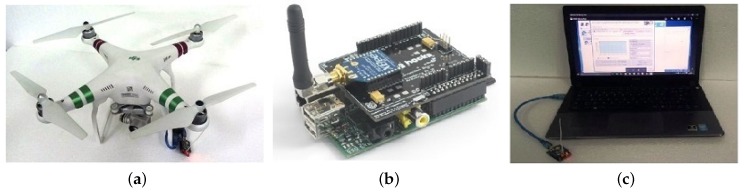
Hardware specification of the proposed multi-UAV system. (**a**) XBee module embedded in UAV Phantom 3 Standard; (**b**) XBee module connected to the power bank; (**c**) BS equipped with a XBee module.

The XBee modules have a range of 6.5 km for a transmission rate of 200 kbps for transporting data in a network IEEE 802.15.4 (ZigBee) with a power consumption of 2050 mW [46]. The BS is a personal computer connected to an XBee device, running the server to exchange messages with the network nodes. To evaluate the network architecture as proposed for the multi-UAV system, a squadron of quadrotor helicopters is employed according to Figure 7. Figure 7a shows the UAV model Phantom 3, while Figure 7b represents the XBee module connected to power bank. Figure 7c corresponds to the BS. The BS employed in the assessed scenarios to validate the FATNET employed in this work acts as the UAV hub in the squadron to sweep the rocket impact area.

Santos et al. [20] presents an aircraft flight and navigation control system using VHF for uplink and S-Band for downlink. There are some advantages associated with ZigBee transmission devices over those using S-band, among the most important ones is the communication range, which is directly related to the operating frequency of the modules. ZigBee devices use a lower operating frequency than S-band, which means less signal attenuation and therefore a greater ability to ensure reliable system communication. Another important advantage is the cost. More sophisticated equipment operating at higher frequencies tends to be more expensive due to increased manufacturing complexity. Finally, another determining factor for choosing ZigBee is that the S-band is restricted to military operations, environmental satellites, TV and other applications. For these reasons, it is feasible to use ZigBee, which is approved by ANATEL (Brazilian National Telecommunications Agency.

#### 4.1.2. XBee Pro 900HP S3B Modules

According to the adopted scanning strategy and the communication flight requirements, module ZigBee IEEE 802.15.4 developed by ZigBee Alliance associated with IEEE protocol was chosen for communication and data routing. This technology has the appropriate characteristics to provide a wireless sensor network with low power consumption [47].

This component allows setting the network in star, mesh and tree topologies. The nodes in a ZigBee network are classified according to their logical functions: coordinator, router and end device. A coordinator is the initial network node, which is always in active mode to perform the network control. The router node is used in the mesh and cluster (tree) topologies to increase the network robustness. The router employs routing tables that allow determining the shortest path for a packet to reach its destination, providing the network with the auto-regeneration features if there is a drop in the functionality of other router nodes. End devices are located at the edges of the cluster and star topologies.

An XBee module is capable of determining reliable routes using an algorithm based on a reactive method derived from an ad-hoc on-demand distance vector (AODV). An associative routing table is used to map a destination node address with its next hop. By sending a message to the next hop address, either the message will reach its destination or be forwarded to an intermediate node, which will route the message on to its destination. If the source node does not have a route to the requested destination, the packet is queued to await a route discovery process. This process is also used when a route fails, which occurs when the source node reaches the maximum number of retries without receiving an ACK [46].

### 4.2. Computer Vision Subsystem

Given a set of images taken from a UAV flying over the ocean with a color camera, the problem consists in detecting unauthorized boats in the area. This process must be executed in real-time conditions and aboard the aircraft. After detecting a boat, the system must send an alert signal to the operator along with an image of the boat.

Silva et al. [48] proposed an algorithm based on a segmentation technique similar to P-Tile as presented in Reference [49], which is associated with morphological operations and machine learning. After acquiring a colored frame candidate, the image is split into three component channels and regions of each channel are selected based on their color when compared to the background. In order to remove the noise caused by small spume regions, morphological operations are applied. The resulting regions are classified as boat candidates. By using this process some spume regions may be wrongly classified as boats. If the boats are not white, it is possible to distinguish them from spume based on the color. However, since most boats are actually white, it is not possible to classify a candidate based only on color.

In order to differentiate boat candidates from spume, a feature descriptor was used. Specifically, a feature descriptor capable of taking into account the shape and texture of the candidate is employed. The histogram of oriented gradients (HOG) feature descriptor described in Reference [50] fits well for this purpose.

**Figure 8:**
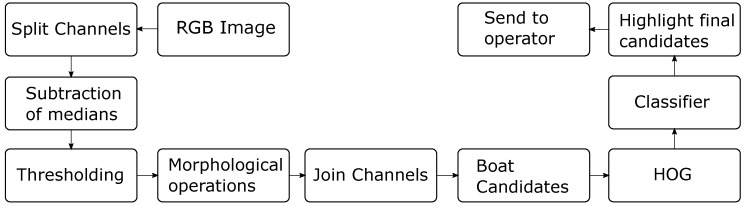
Sequence of steps of the proposed algorithm.

Once the HOG feature vector is calculated for each boat candidate, it is possible to use classification techniques to distinguish between a boat and a spume. Supervised machine learning techniques were adopted for this purpose, that is, Support Vector Machine (SVM), *K* nearest neighbor (KNN) and decision trees, which were trained with a set of labeled images for classifying new images. The complete schematic of the proposed system is shown in Figure 8.

### 4.3. Software Architecture

In order to enable proper information exchange, a system was developed using the client server model capable of exchanging messages between the network nodes and the BS. The communication requirements of the application include the transmission of images processed by the computer vision subsystem; text messages to obtain the aircraft position; the chronology of events; verification of the transmitted data integrity and confirmation of received data. The software is designed to meet the requirements imposed by the image processing subsystem and operate on a network composed of XBee sensors. This architecture can be used in other communication protocols or network architectures for UAV area scanning, where it is necessary to fragment and send data safely to ensure the receipt of all packets. Based on the aforementioned requirements, the system has three types of messages:

- Type 1: control messages (CtrM), which are periodically transmitted from the network to the BS containing data on telemetry and the swept area;
- Type 2: data messages (DM), which are scanned transmitted from a UAV to the BS when a boat is found, containing the image of the target;
- Type 3: confirmation messages (CnfM), which correspond to the response sent from the BS to a UAV to confirm the receipt of a data message.

#### 4.3.1. Control Messages

CtrMs contain command and telemetry information obtained from the UAVs, allowing all components of the multi-UAV system to identify the aircrafts that compose the mission, their respective geographic positions and the eventual position of boats. Messages are sent periodically, that is, every three seconds to the BS. The CtrM format is described in Table 2 and a description of the fields that constitute the header is presented as follows.

- Identification: unique identifier of the message within a sequence. The packet starts with zero value, while the following ones are incremented by one unit until the mission is finished;
- Message Type: it indicates the packet type, for example, type 1 in this case;
- Source Node Address: identifier of the node that generated the message;
- Destination Node Address: identifier of the node to which the message is addressed;
- Packet Number: unique identifier of a packet sequence. All packets that belong to a same sequence assume a constant value for this field. This field is used in the reassembly process. A node must never assign the same number to a different sequence;
- Time: it forms the date and time of the packet origin using Unix timestamp format;
- Current Location: it contains the geographic coordinates of the source node;
- Number of targets found: this parameter is initialized with zero, being incremented by one unit when a boat is detected;
- Location of found targets: it contains the geographic coordinates of possible targets.

The CtrMs are intended to provide information to the operators in the BS on the area scanned by the UAV squadron, which includes the eventual location of a boat. The "location of targets" field is cumulative, being responsible for sending the coordinates of the detected target and the location of the previous target if it exists. Thus, when the imaging system detects a possible boat, the last two geographic coordinates remain in subsequent packets in such a way that if the packet is lost for some reason, the next ones continue to send information on the location of the target found. In order for the control messages to be of the same size, the fields are filled with zeros if any boats have not yet been found.

#### 4.3.2. Data Messages

DMs are used to transmit images of a detected boat from the UAV that found the target. The DM format is presented in Table 3 and a description of the fields that constitute the header is given as follows:

- Identification: unique identifier of the message within a sequence. The initial packet starts with zero value, while the following ones are incremented by one unit until the value of the last packet is reached;
- Message type: it indicates the packet type, that is, type 2 in this case;
- Number of packets: it indicates the number of packets that must be sent;
- Source Node Address: identifier of the node that found the boat and generated the message;
- Packet Number: unique identifier of packet sequence, whose value is equal to the field of the CtrM header that contains the boat location. All packets that belong to a same sequence assume a constant value for this field. This field is used in the reassembly process, being associated with the source node address to identify the target location;
- Data: it contains the fragmented image of the target.

#### 4.3.3. Confirmation Messages

CnfMs transmit the receipt acknowledgment from the receiver (BS) to the transmitting UAV. The CnfM sends only the confirmation of the last received packet through the identification field. If any data message has not been received, it requests the retransmission of the IDs that were not received from the ID number.

The CnfM format is presented in Table 4 and a detailed description of the fields that constitute the header is given as follows:

- Identification: identifier of the last received packet;
- Message Type: it indicates the type of the packet, that is, type 3 in this case;
- Destination Node Address: identifier of the node that localized the boat;

#### 4.3.4. Operation of the Multi-UAV System

**Figure 9:**
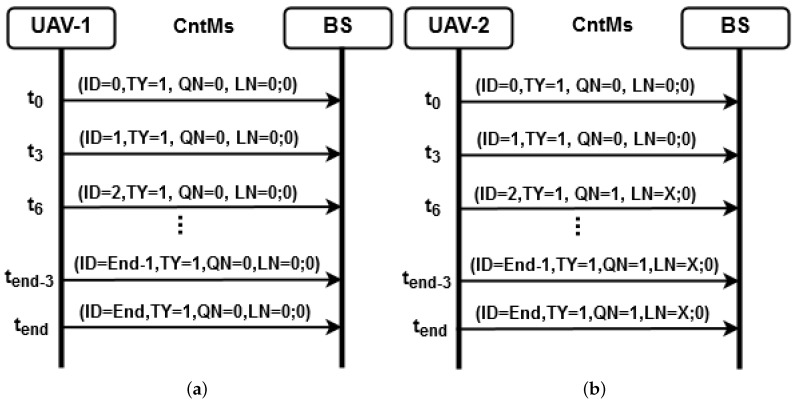
Flow of CntMs to the BS in a FANET: (**a**) UAV-1 completed the mission without detecting boats; (**b**) the imaging system located a boat, while informing the BS and preparing a DM.

CntMs are sent by all the UAVs that constitute the FANET to the BS periodically. The message flow is shown in Figure 9. In this scenario, the BS receives information on the area from each VANT and whether any boat is found. At the initial time instant t${}_{0}$, each UAV sends a CntM reporting the scanning situation, while also transmitting a new message every three seconds until the mission ends. Figure 9a represents the scanning scenario of UAV-1, where the mission was completed and no boat was found. Figure 9b shows that UAV-2 found a boat at instant t${}_{6}$ in the coordinate X, thus informing the BS about the number of boats located so far. From this event, UAV-2 starts sending a DM to transmit the image of the detected target to the BS, while scanning continues and new CntMs are sent. When the server located at the BS receives a CntM indicating the target location, it informs the operator that a boat was found at a given geographic coordinate, as a sound alert can even be emitted if necessary.

**Figure 10:**
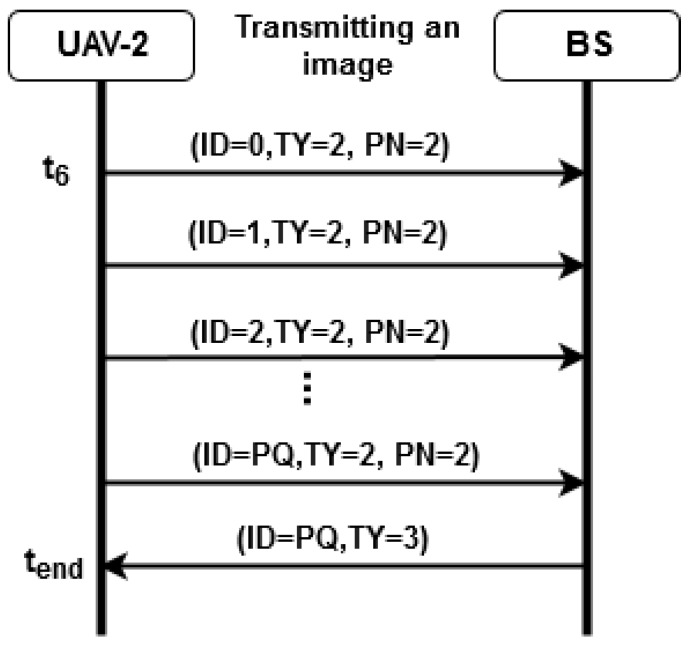
Message flow for transmitting an image.

The image transfer process is explained in Figure 10. After locating a boat, UAV-2 starts sending the image to a BS using a type-2 DM. At instant t${}_{6}$, UAV-2 starts the image transmission through a given number of DMs defined according to the value of PQ. It is observed that the ID field contains the position of each message. Besides, the last one will contain an ID equal to PQ. Once the receipt has been confirmed, the BS checks if all messages have been received correctly. In this case, a type-3 CnfM is sent to confirm that retransmission is not necessary since ID is equal to PQ. Otherwise, the message payload is supposed to contain the message IDs required for reassembly of the original data.

## 5. Description of the Scenarios for FANET Test

In order to evaluate the network performance in the multi-UAV system, a squadron of quadrotors and a BS were assembled. Some modules were embedded in UAV: an XBee Pro 900HPS3B module connected to an XBee Explorer USB module and a power module (Power Bank). The BS is composed of a laptop with communication and test software, while the XBee module is connected to the XBee Explorer USB module.

During the performed tests, the information is transmitted from the FANET to BS. A laptop performs U2S communication, being responsible for collecting data on the performed simulations. In the multi-UAV system used in CLBI, this node assumes the function of the UAV hub. While the BS corresponds to the GCS in the tests, the UAV hub connects the GCS to the network composed of aircrafts when scanning the rocket impact area.

The spiral scanning strategy with constant flight speed was adopted without considering area division. The distance for which the nodes have reliable communication was defined through simulation of the maximum range between UAVs. The tests allowed to analyze the time interval required to reorganize the network in case of node loss were analyzed. For this purpose, the latency, transfer rate, received packet rate and signal strength during data transmission was compared in several scenarios.

**Figure 11:**
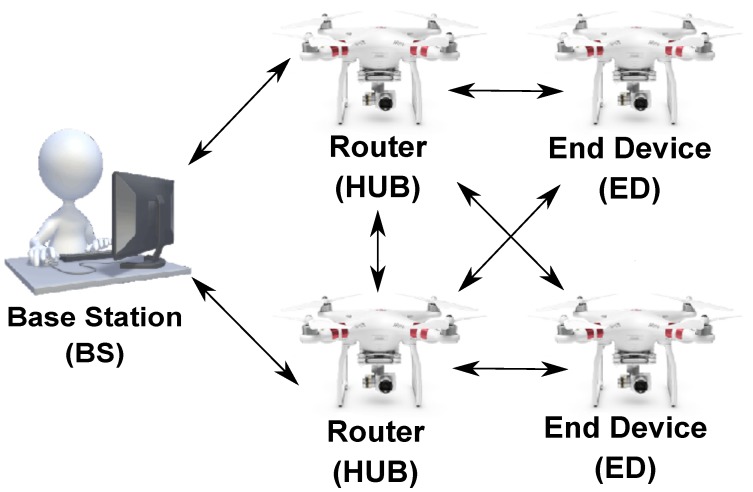
ZigBee devices in a scenario FANET test with mesh configuration.

The exchange of messages between the BS, which is referred to as ZigBee Coordinator Node (CN) and the HUB UAV constitutes a peer-to-peer network. The XBee modules in the UAVs are configured as ZigBee routers (HUB) and ZigBee End Device (ED), corresponding to a mesh network capable of sending data to the coordinator node which is responsible for forwarding messages from the squadron to the BS and vice-versa, shown in Figure 11. The objective is to evaluate the performance and reliability of the network through data transmission tests, as well as the reconfiguration capability case of the HUB node failure.

X-CTU^®^ free software was used for network management and monitoring [51]. The test and analysis of performance metrics, for example, RSSI, delay time (latency), transmission rate (link speed) packet loss rate and overhead were executed. The transmission of CtlMs and DMs with known and fixed size can then be simulated. Data transmitted in the network can be properly controlled, avoiding packet fragmentation in lower layers.

**Figure 12:**
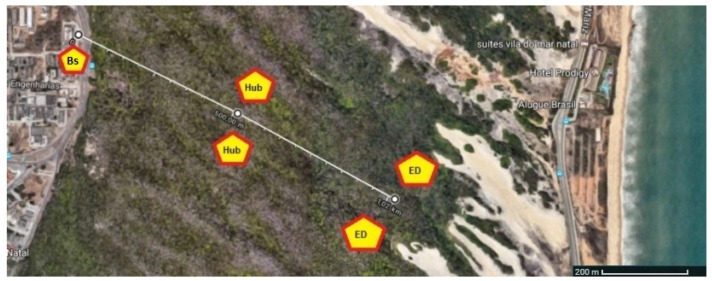
Test site and position of UAVs.

The tests were performed with the BS located at the central campus of the Federal University of Rio Grande do Norte, Natal, Brazil, while the UAVs traveled over the sand dune park in Natal, which is a region with a constant wind regime and almost no electromagnetic interference. The tests were executed late in the afternoon during periods with few clouds. Messages were sent with different transmission rates within the limit supported by XBee. In order to simulate the transmission, a 100-kB image was used to detect boats as described in Santos et al. [20], where 500 packets of 256 bytes were transmitted. Figure 12 shows the test site.

### 5.1. Scenario 1—Baud Rate of 115,200 bps

This scenario simulates the situation where a boat is found by a FANET node. There is no failure of any node. In order to validate the network operation, 500 packets of 256 bytes were sent. This procedure was repeated 72 times with the aim to calculate the mean and standard deviation of the samples. Packet sending time, packet loss, signal strength (RSSI) and throughput were obtained.

**Figure 13:**
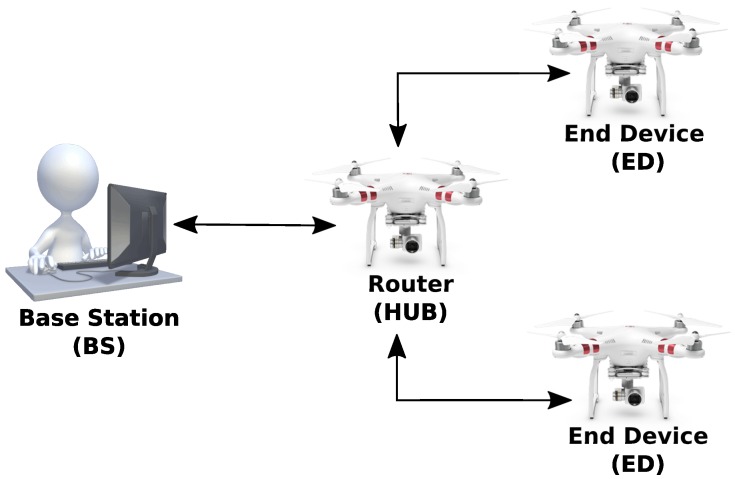
ZigBee devices in a scenario 1 with mesh configuration.

In this configuration shown in Figure 13, the BS and the network coordinator were on the ground. The first UAV was added to the network as a router (HUB), flying at a height of 80 m from the ground. Its respective position carried from 400 m to 600 m away from the BS. The second UAV was added as an end device, flying at a height of 80 m from the ground, with the position varying between 900 m and 1100 m from the BS. The baud rate for all XBee modules was set at 115,200 bps as listed in Table 5.

### 5.2. Scenario 2—Baud Rates of 38,800 bps and 115,200 bps

**Figure 14:**
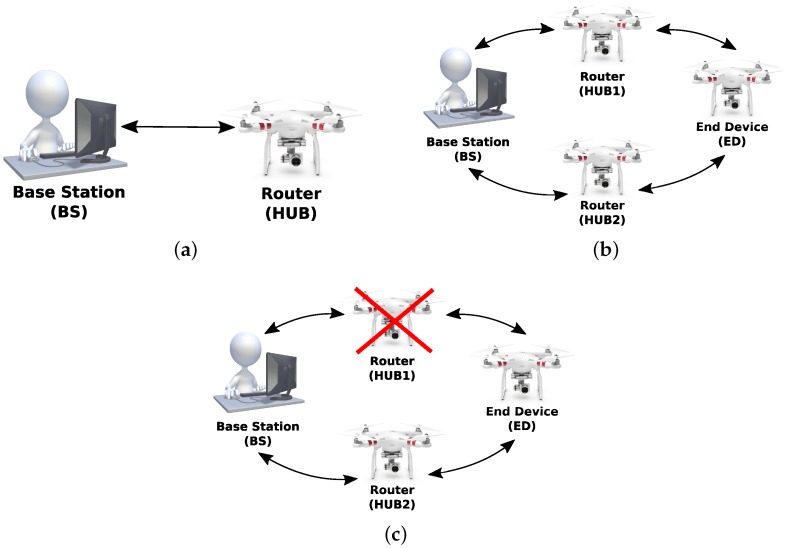
ZigBee devices in configuration scenario 2. (**a**) ZigBee devices in P2P configuration scenario; (**b**) ZigBee devices in a scenario without failure; (**c**) ZigBee devices in a scenario with failure.

According to Figure 14, this scenario simulates the situation where a HUB node fails and exits the network during the transmission of image data, forcing the nodes to be reorganized, searching for an alternate route (i.e., another hub) to continue the packet transmission between the ED and the BS. The XBee Modules were configured in such a way that the ED did not have a direct link to the BS. Thus, the transmitted packets always hop through a HUB. In this configuration, when the HUB is deactivated, the ED must find an alternative route through another HUB to send packets, since it is not able to transmit packets directly to the BS, that is, without the hop. In order to simulate this scenario, the baud rates of the ED and BS were set to 38,800 bps, while the baud rate of the HUBs was 115,200 bps.

In this new arrangement, the coordinator was on the ground again. However, in this case, two UAVs work as routers (HUBs) maintaining their formation close to each other. The first and second UAVs flew at altitudes of 70 m and 80 m, respectively, with a distance varying from 400 to 600 m from the BS. A third UAV (ED) was added to the network as the end device, flying 80 m high and distant 900 to 1110 m from the BS. This condition is described in Table 6.

## 6. Results

### 6.1. Antenna Modeling and Characterization

This section aims to define a position to place the XBee module in the UAV so that the antenna lobe has minimal deformation caused by the aircraft structure as stated in Reference [52] where it is effectively shown that the aircraft structure can cause normal log shading. This effect can be harmful to communication and has been minimized by using 4nec2 software [53] to determine the location of the XBee module antenna in the UAV. The antenna model and the definition of its position in the aircraft are discussed in this section.

During the flight, the XBee device was attached to the aircraft structure. One of the main concerns was to ensure that the UAV would be able to send data regardless of the direction it was flying to. For this purpose, the antenna was positioned so that the internal and external metallic structures of the aircraft could not interfere with the antenna radiation lobe. The XBee antenna is omnidirectional with an approximate gain of 2 dBi.

In order to analyze the structure in 2D and 3D view from the antenna, 4nec2 software [53] was used. This software is capable of comparing distinct radiation patterns of distance field, as well as verifying the voltage standing wave ratio (VSWR) and gain through frequency sweeping. A filament antenna was created with the same characteristics obtained from the XBee datasheet to simulate the metallic structure used by the aircraft. The antenna was positioned at different places of the UAV to reach a minimum interference.

**Figure 15:**
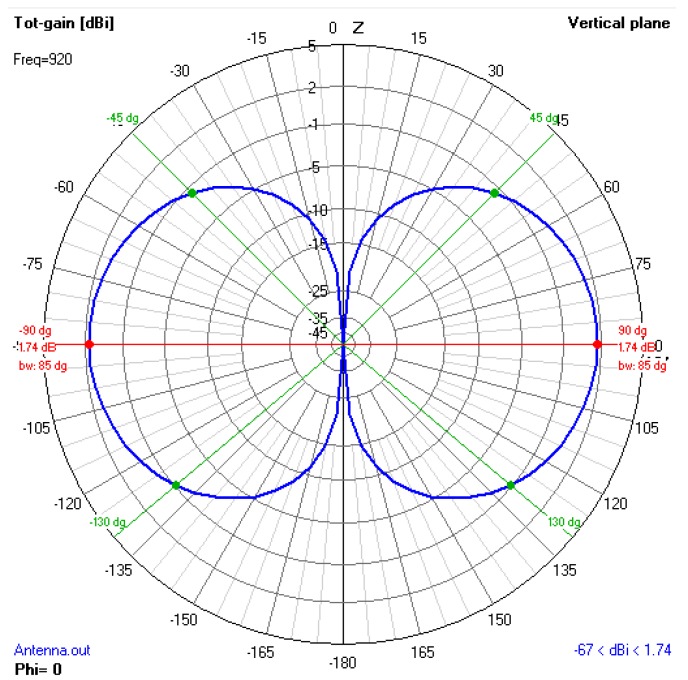
Analysis of the best position of XBee antenna by simulation using 4nec2 software.

**Figure 16:**
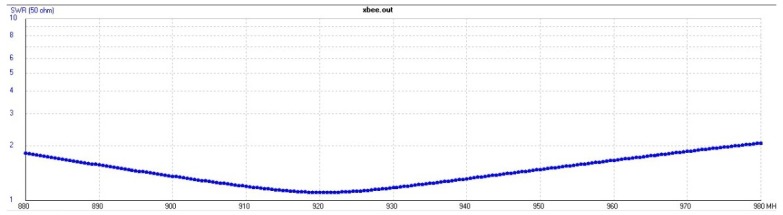
Simulation results of the operating frequency range of XBee antenna.

It can be stated that keeping the antenna at a distance greater than or equal to λ/4 from the metallic structure leads to good results since low interference is obtained as seen in Figure 15 and Figure 16. Figure 15 shows that the vertical lobe presents omnidirectional irradiation with a maximum edge gain of 1.74 dBi and a half-power beamwidth with 90°. The green lines represent the half-power beamwidth and the red line indicates the position of maximum gain of the antenna. Considering the XBee antenna, the half-power beamwidth is around 85° [54].

Figure 16 represents the VSWR coefficient, whose ideal value is close to unit. If this condition occurs, the impedance match between the transceiver and antenna will be more efficient at the maximum rated power. Therefore the antenna operating frequency will not change with the distance when compared with the datasheet specification.

### 6.2. Simulation of the Maximum Range between UAVs

**Figure 17:**
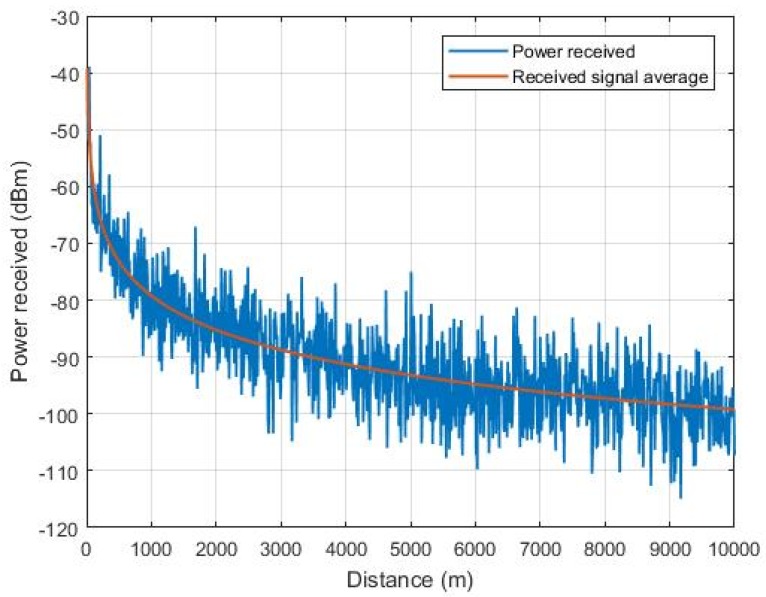
Simulated behavior of the power received by the XBee transceiver.

The effects caused by the flight environment, for example, buildings in urban areas or vegetation suburban areas lead to flight distance restrictions due to the Doppler effect caused by aircraft movement. Therefore multipath caused by local spreaders and the natural weakening of the signal due to the distance are of major concern. The manufacturer in Reference [46] states that Xbee devices can communicate at a distance of up to 6.5 km. Figure 17 presents the simulation of the received power considering the log-normal shading effect. Through the simulation results, due to QoS issues, it was determined that the maximum distance may not exceed 5 km, since the receivers have a maximum sensitivity of −100 dBm. This distance was determined based on Equation (4). Figure 17 shows the simulation of the received power versus distance profile with a standard deviation of 6 dB based on Reference [55], as well as the average received power. This equation corresponds to a simplified model for large-scale fading (path loss and shading) and does not consider the Doppler effect. Table 7 shows the simulation parameters.

Then, an analysis of the aircraft-embedded XBee network performance is made when measuring latency, transfer rate, number of received packets and signal strength. The input parameters for the calculation of the received power are shown in Table 7.

### 6.3. Experimental Results

Throughput and RSSI tests were performed in scenarios 1 and 2 in order to verify which configuration is more efficient for structuring the network. In these conditions, transmission speeds with different baud rates were measured. The throughput was calculated based on the simultaneous bidirectional packet transmission between nodes. To increase the reliability of results, the test was repeated 72 times in scenario 1. In scenario 2, three different experiments were performed, each one of them repeated 36 times while sending 500 bidirectional packets per test.

#### 6.3.1. Scenario 1

In scenario 1, 72 experiments were performed and packet loss occurred in seven cases, resulting in 90.78% of tests without losses. In such cases, the total amount of lost packets was equal to 15, where four packets are the maximum number obtained in a single experiment. 500 packets per experiment were sent, comprising a total of 36,000 packets, where 99.96% of the transmitted packets were successfully received. The average throughput was 15.56 kbps, with an average transmission time of 67 s and local and remote RSSIs equal to −50.00 and −50.00 dBm, respectively. The best result was obtained in 62 s, where no packets were lost. The aforementioned results are summarized in Table 8 and Table 9.

#### 6.3.2. Scenario 2

In scenario 2, three different configurations were set to evaluate the performance:

- first case: the peer-to-peer transmission between ED and BS was performed, shown in Figure 14a. There was no packet hop through a multi-hop network. The distance between ED and BS nodes was less than 800 m.
- second case: the transmission was carried out with a hop through a hub. The position of the nodes was the same as that in scenario 1, shown in Figure 13.
- third case: the configuration was the same as that in case 2, shown in Figure 14b but the hub node that routed the packets was deactivated during transmission, thus exiting the network, Figure 14c.

In the first case with peer-to-peer configuration, 36 experiments were performed and packet loss was verified during 15 of the existing sessions, that is, 58.33% of the tests did occur without loss. 17 packets were lost during the remaining tests, where 99.90% of the transmitted packets were received. The average throughput was 8.92 kbps, with an average transmission time of 116 s and average local and remote RSSIs of −49.40 and −51.20 dBm, respectively.

In the multi-hop configuration corresponding to the second case, 36 experiments were performed and packets were lost during only two ones. In other words, 94.44% of the performed tests did not present packet loss. In the remaining experiments, only two packets were lost, that is, 99.98% of the transmitted packets were successfully received. The average throughput was 7.48 kbps, with an average transmission time of 138 s and average local and remote RSSIs of −50.88 and −50.92 dBm, respectively.

In the last case using multi-hop failure configuration, 36 experiments were also performed and packets were lost during 30 sessions, that is, 16.67% of the tests were carried out without loss. Among the remaining experiments, the total amount of lost packets was 31, that is, 99.82% f the transmitted packets were successfully received. The average throughput was 7.13 kbps, with an average transmission time of 143 s and average local and remote RSSIs of −51.24 and −50.86 dBm, respectively. In all experiments, the connection could be successfully reestablished. The aforementioned results are summarized in Table 10 and Table 11.

### 6.4. Analysis and Comparison of Results

Baud Rate and Throughput: In scenario 1, all nodes have a baud rate set to 115,200 bps. Throughput is higher, with an average value of 14.77 kbps. In scenario 2, the throughput decreases when the packets hop through the hub. This is due to the additional time interval required by the intermediate node to receive a packet, process its respective internal routing table and transmit it to the next node. The throughput without and with hop was measured as 8.92 kbps and 7.48 kbps, respectively, being reduced by 16.14%. The results are summarized in Table 12, which are compared with the ones obtained during tests in scenarios 1 and 2.

Latency can be properly analyzed in scenario 2. The average difference in the transmission time between mesh and peer-to-peer topologies was 22 s. Nevertheless, more than 94% of the images were successfully transmitted between two nodes, when a hop through an intermediate node occurred. No more than 59% of the images were transmitted without any loss when an intermediate node was not available. The mesh topology network is more reliable due to the redundancy in terms of existing paths and the short distance between the nodes. The delay is due to the processing time of a hop.

When hub failure occurred, the percentage of tests without packet loss was 16.67%. This value may give the impression that the network is unreliable but two factors must be considered. Firstly, in the particular case of a UAV compact formation, the failure of a node is an unlikely event. Secondly, when a node exits the network, the packet under processing at that very moment will be necessarily lost. Therefore, in this specific case, it is important to analyze if the network is capable of reorganizing itself, how long this procedure takes to occur and the number of packets that may be lost during reconfiguration. According to Table 12, in the case of a node failure, the average transmission time was five seconds longer when compared with operation under normal conditions. Among the 30 tests with hub failure where the loss occurred, a single packet was lost during 28 tests. During the other two sessions, four and five packets were lost. There is not a proper explanation for which there was no packet loss in the remaining six tests with hub failure. Possibly this is due to the fact that the node did not transmit data during the failure. It is also worth mentioning that the network found an alternate path and remained operational in 100% of the cases where a node failed.

In general, the experiments have shown that the network is reliable. The mesh topology has proven to be a more appropriate choice than a point-to-point topology for a multi-UAV system. Analyzing the results, it can be stated that the ideal configuration lies in using a baud rate of 115,200 bps, with two or more nodes configured as routers to ensure route redundancy and the possibility of defining alternate paths form the terms and DMs to be transmitted to the BS.

## 7. Conclusions and Future Work

This work has presented results on the specification of a communication network architecture for a multi-UAV system for scanning the impact area of rockets launched from CLBI in order to detect invasive boats in an exclusion zone established during the launch chronology. The main features of the mobile FANETs have been properly addressed. Despite recent advances in studies regarding communication networks applied to multi-UAV systems, it has been demonstrated that there is no general network architecture that meets the requirements and characteristics of all existing applications.

In the specific case of applications associated with scanning the rocket impact area, which have very specific characteristics such as propagation and attenuation of radiofrequency signals on the sea surface, the challenge is even greater. A hardware and software architecture to support the communication network is currently incorporated into a squadron. With such infrastructure, experimental tests will be carried out to validate the proposed approach.

Tests were performed using the scanning strategy without area decomposition. In order to validate the proposed strategy, a network architecture based on ZigBee technology was defined. For this purpose, different scenarios and configurations were adopted to analyze the network performance when transmitting a 100-kB image. The network presented stable behavior while transmitting the image with a baud rate of 115,200 bps within an average time interval of 67 s. During 100% on the test where the failure occurred, the results clearly denote that the network is capable of reorganizing itself and maintaining data transmission with success rates higher than 99%. Since in such type of application the FANET is required to transmit an image with reliable data to confirm the detection of a boat even when data transmission fault occurred, the experiments carried out in this work allow concluding that the network is a reliable fault-tolerant, fulfilling the necessary requirements satisfactorily.

The next steps of the project include additional tests to increase the amount of data, as well as the development of a hardware and software architecture associated with interpixel redundancy for image reconstruction in cases of packet loss.

## Figures and Tables

**Table 1:** Characteristics of the Multi-UAV communication network for the proposed scanning strategies.

Characteristics	Without Subdivision of the Area	With Subdivision of the Area
Formation	Close nodes as function of the field of view of the camera	Distant nodes as a function of the radio range
Required time interval for scanning the area	Proportional to the number of the nodes	Proportional to the number of the nodes
Size of the monitored area	Limited by the radio range of the UAV hub	It can be extended from the UAV out of range through the mesh network
Type of communication\routing	Star or Mesh, with little design modification	Mesh
Topology of the network between the UAVs (nodes)	Start or Mesh	Mesh
Topology of the network between UAVs and base station	Star, need of a hub node to communicate with the base station over the entire area	Mesh, wider range of communication
Distance between nodes	Short	Long
Required time interval for interruption in case of node failure	Short	Long
Reorganization in case of failure node	Low operational cost independent of the scanning method	Medium and high cost when using the back-and forth and spiral method, respectively
Energy consumption	Low in the case of end devices	Medium/high in routers and coordinators
Type of node	Star Topology: one or two coordinators, one router end devices required	One or two coordinators and routers required
Application	Scanning time is the major concern, thus requiring a node with higher range and processing capacity.	Larger scanning areas. The nodes have the same transmission and processing capacity.

**Table 2:** Control message (CtrM) Format.

Field		Size (bytes)
ID	Identification	4
TY	Message Type	1
SN	Source Node Address	2
DN	Destination Node Address	2
PN	Packet Number	4
TI	Time	10
LA	Current location	18
QN	Number of targets found	4
LN	Location of targets	36

**Table 3:** Data message (DM) format.

Field		Size (bytes)
ID	Identification	4
TY	Message Type	1
SN	Source Node Address	2
PQ	Number of Packets	4
DN	Destination Node Address	2
PN	Packet Number	4
DA	Data	239

**Table 4:** Confirmation messages (CnfMs) format.

Field		Size (bytes)
ID	Identification	4
TY	Message Type	1
DN	Address Destination Node	2

**Table 5:** Scenario 1: Configuration and position nodes.

Parameter	BS	HUB	ED
ZigBee Function	Coordinator	Router	End Device.
Distance from BS	-	400–600 m	900–1100 m
Baud Rate	115,200 bps	115,200 bps	115,200 bps
Sent Bytes	128,000	-	128,000
Sent packets	500	-	500

**Table 6:** Scenario 2: Configuration and position nodes.

Parameter	BS	HUB	ED
ZigBee Function	Coordinator	Router	End device
Distance from BS peer-to-peer	-	-	700–800 m
Distance from BS with hop	-	400–600 m	900–1100 m
Baud Rate	38,800 bps	115,200 bps	38,800 bps
Sent Bytes	-	-	128,000
Sent Packets	-	-	500

**Table 7:** Frequencies and respective impedances.

Parameter	Value
PTx—trasnmission power	23 dBm
GTx—transmission antenna gain	1.7 dBi
GRx—receiving antenna gain	1.7 dBi
λ—wavelength	0.33 m
$X_{\sigma }$—random variable	6.0 dB

**Table 8:** Results obtained in scenario 1.

	Received Bytes	Sent Time (seconds)	Local RSSI (dBm)	Remote RSSI (dBm)	Tx (kbps)	Received Packets	Lost Packets
Average	127,925	01:07	−50	−50	15.56	499.7	0.3
Best Result	128,000	01:02	−48	−47	16.38	500	0
Worst Result	126,976	01:36	−49	−54	10.56	496	4
mean deviation	237	00:10	2.79	2.79	1.63	0.93	0.93

**Table 9:** Analysis of results obtained in scenario 1.

Parameters	Results
Number of tests	72
Tests without lost packets	90.28%
Received bytes	99.96%
Average Throughput	15.56 kbps
Average time to send image	67 s

**Table 10:** Results obtained in scenario 2.

	Received Bytes	Sent Time (seconds)	Local RSSI (dBm)	Remote RSSI (dBm)	Tx (kbps)	Received Packets	Lost Packets
Peer-to-peer	127,872	01:56	−49.4	−51.2	8.92	499.5	0.5
Multi-hop	127,974	02:18	−50.9	−50.9	7.48	499.9	0.1
Multi-hop with failure	127,770	02:23	−51.2	−50.8	7.13	499.1	0.9

**Table 11:** Analysis of results obtained in scenario 2.

Parameters	Peer-to-Peer	Multi-Hop	Multi-Hop with Failure
Number of tests	36	36	36
Tests without lost packets	58.33%	94.44%	16.67%
Received bytes	99.90%	99.98%	99.82%
Average Throughput	8.92 kbps	7.48 kbps	7.13 kbps
Average time to send image	116 s	138 s	143 s

**Table 12:** Results of data transmission tests. * Network reconfiguration test performed only in scenario 2 in the third case (02 Multi-hop with failure).

Scenario	RSSI Average (dBm)		Average Throughout (kbps)	Average Transmission Time (seconds)	Tests without Loss of Packets (%)	Received Packets (%)	Network Reconfiguration after Failure (%) *
	Local	Remote					
01	−50.00	−50.00	14.77	67	90.78	99.96	-
02 P2P	−49.40	−51.20	8.92	116	58.33	99.90	-
02 Multi-hop	−50.88	−50.92	7.48	138	94.44	99.98	-
02 Multi-hop with failure	−51.24	−50.86	7.13	143	16.67	99.82	100
